# When the introvert stands out

**DOI:** 10.1016/j.isci.2024.108989

**Published:** 2024-02-15

**Authors:** Azana Marie Cochran

**Affiliations:** 1 Michigan State University Fisheries and Wildlife, Michigan State University, 711 West Grand River Avenue, East Lansing, MI 48823, USA

## Abstract

RBSA Honorable Mention.

## Main text

**Figure:**
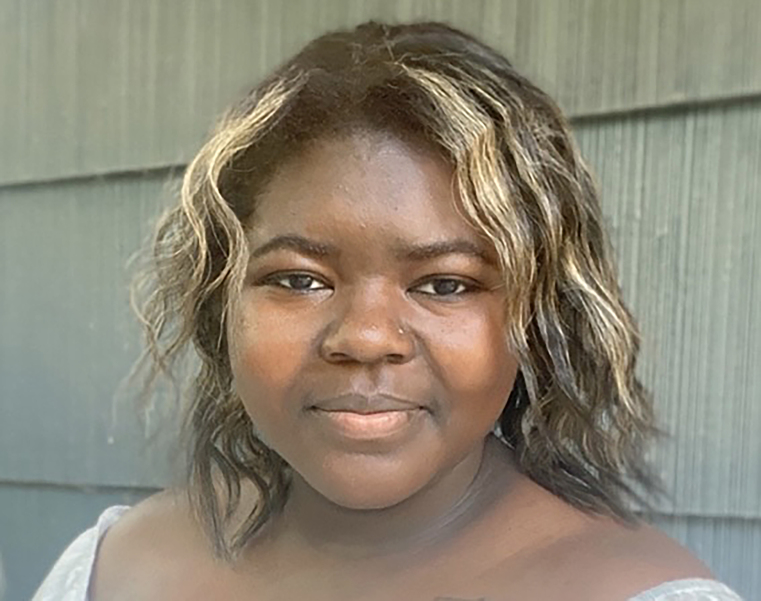
Above image: Azana.

Being in and around nature has been one of the few things that have stayed a constant in my life from childhood to adulthood. My grandparents were the first ones to introduce me to what the environment can offer. With them being farmers my days were mainly spent outside walking through forest trails, trying to identify plants, and spending time with the animals. Being accustomed to this lifestyle from such a young age, I believed that no human interaction could ever match the freedom I felt to be fully myself with nature and animals. Nature does not discriminate or hold bias; animals gravitate toward a person’s presence not their skin color. Ultimately, that led to the development of my introverted persona. Never fully allowing outsiders to know me or take risks. This only intensified when being one of few Black people in school or activity became a norm. I figured if I just kept my head down, stayed quiet, and do as I was told, I could just blend into my surroundings. Maybe then people wouldn’t notice my Blackness and how I stick out among most of my peers.

During my freshman year in undergraduate I accepted a summer position in Cleveland, Ohio researching the behavioral boldness of rural versus urban coyotes when they were in contact with a foreign object. This was my first of many amazing experiences being able to lead a research project and learn the skills needed to become a great biologist. But it wasn’t hard to notice the lack of diversity throughout the wildlife conservation world. It was so obvious people closest to me would comment how being a wildlife biologist was a “white man’s” job. They would purposely point out the fact that I would the “only” or “first” Black person in a dominated white space. Being the first or only of anything in an introvert’s mind is already a very uncomfortable space that one tends to avoid. Especially since I had spent most of my youth trying to hide my Blackness.

I have been very fortunate to work in a variety of different labs through my time at university. I have worked alongside graduate students on sea lamprey pheromones and genetics, and how that impacts populations in The Great Lakes. I have also worked with professors on conservation efforts internationally in Pakwach, Uganda on an initiative called Snares to Wares. This initiative helps provide solutions for human-wildlife conflict through snare wire poaching and improve community livelihood. The most memorable experience, though, was being an intern at a zoological facility doing captive cougar behavioral work as well as outreach and tours. During the end of one tour, I had young Black girl and her mother come up to me to ask about the animals, conservation, and personal questions about myself. The longer I talked with this young girl, the more I saw her face light up with excitement and interest. What stuck with me the most was when this girl told me she wanted to be just like me and help save the animals. As someone who never saw someone who looked like me in conservation, this comment made me challenge so many of my fears. I believed that I was the type of person to blend in, not stand out. Follow orders, not be a leader. If I took this career path into wildlife research, I would have to acknowledge and be comfortable being one of very few minorities, at least for now.

Now as I finish up my last year, I have grown in ways I could never thought possible. Though still an introvert, I have been able to find my confidence in being a leader in cutting edge conservation research. Currently, I am an intern with the United States Fish and Wildlife Service (USFWS) to assist in research efforts on bald eagle genetic diversity within the Great Lakes. I also assist other biologists to research the impact of contaminants within Michigan rivers and lakes on bald eagles, freshwater mussels, and other water-reliant species. If the younger version of myself could see me today, she wouldn’t be able to believe this is what we have become. No longer trying to hide behind the shadows and embracing the beauty of being a Black woman. For the future, I will continue in my studies to graduate school and continue working with USFWS to continue research on threatened and endangered species. I won’t just stop at research though; I want to lead the efforts to make strides in diversifying the field of wildlife conservation. Being the first or only is scary, but someone must do it to pave the way for others, and I will gladly take on that challenge.

